# Exploiting Human Resource Requirements to Infer Human Movement Patterns for Use in Modelling Disease Transmission Systems: An Example from Eastern Province, Zambia

**DOI:** 10.1371/journal.pone.0139505

**Published:** 2015-09-30

**Authors:** Simon Alderton, Jason Noble, Kathrin Schaten, Susan C. Welburn, Peter M. Atkinson

**Affiliations:** 1 Institute of Complex System Simulation, School of Electronics and Computer Science, University of Southampton, Southampton, United Kingdom; 2 Geography and Environment, Faculty of Social and Human Sciences, University of Southampton, Southampton, United Kingdom; 3 Division of Pathway Medicine and Centre for Infectious Diseases, School of Biomedical Sciences, College of Medicine and Veterinary Medicine, The University of Edinburgh, Edinburgh, United Kingdom; 4 Faculty of Science and Technology, Engineering Building, Lancaster University, Lancaster, United Kingdom; 5 Faculty of Geosciences, University of Utrecht, Heidelberglaan 2, 3584 CS Utrecht, The Netherlands; 6 School of Geography, Archaeology and Palaeoecology, Queen’s University Belfast, Northern Ireland, United Kingdom; Université Toulouse 1 Capitole, FRANCE

## Abstract

In this research, an agent-based model (ABM) was developed to generate human movement routes between homes and water resources in a rural setting, given commonly available geospatial datasets on population distribution, land cover and landscape resources. ABMs are an object-oriented computational approach to modelling a system, focusing on the interactions of autonomous agents, and aiming to assess the impact of these agents and their interactions on the system as a whole. An A* pathfinding algorithm was implemented to produce walking routes, given data on the terrain in the area. A* is an extension of Dijkstra’s algorithm with an enhanced time performance through the use of heuristics. In this example, it was possible to impute daily activity movement patterns to the water resource for all villages in a 75 km long study transect across the Luangwa Valley, Zambia, and the simulated human movements were statistically similar to empirical observations on travel times to the water resource (Chi-squared, 95% confidence interval). This indicates that it is possible to produce realistic data regarding human movements without costly measurement as is commonly achieved, for example, through GPS, or retrospective or real-time diaries. The approach is transferable between different geographical locations, and the product can be useful in providing an insight into human movement patterns, and therefore has use in many human exposure-related applications, specifically epidemiological research in rural areas, where spatial heterogeneity in the disease landscape, and space-time proximity of individuals, can play a crucial role in disease spread.

## Introduction

### Risk, exposure and human movements

Humans are exposed to a variety of hazards as part of their everyday routines which, in turn, carry associated risks [1].

Whether the hazard is physical degradation [2], disease-bearing flies [3], or chemical toxins [4], a high degree of exposure can result in considerable risk to human health and wellbeing [5]. Unfortunately, a degree of exposure to such hazards is often unavoidable in order to obtain the resources upon which humans depend. Indeed, examples range from the financial resources obtained from attending work which potentially exposes people to influenza, to collecting basic water resources from a river surrounded by mosquito- or tsetse fly-infested bush. Given that exposure is at the heart of this problem, the study of human movements can provide important information on how exposure to such hazards varies through time and space, with potential implications in policy making and risk mitigation [6].

Gaining an insight into the dynamics of a disease can be crucial in developing forms of control and mitigation, whether this is reducing the impact of nodes or hubs which exhibit high infection rates, or disconnecting areas to limit infection pathways. One means of gaining this understanding of disease dynamics is the investigation of human movements relative to pathogen prevalence and host abundance, whether these are migratory flows over large geographical areas [7, 8], or daily movement routines of individuals within or between local settlements [9]. Although the measurement of human movements is primary, and can be captured through the use of, for example, mobile phone data [10], GPS, and individual diaries, these data are not always readily available or accessible, due to a lack of human or financial resources, or possible privacy concerns. Furthermore, the lack of available literature which considers individual human movement patterns has been highlighted [9].

Modelling human movement patterns is an alternative approach to direct measurement, and literature exists which considers ‘activity spaces’, including patterns concerning time allocations at different locations and frequency of these trips from a home site [11, 12]. Although this helps gain an understanding of people’s routines and, therefore, of periods when people might be exposed to hazards due to their location, often the journey itself is not modelled in detail. For urban settings, indoor or in-vehicle activity spaces are primary. However, where the hazard occurs outdoors, and given the degree of spatial heterogeneity in some rural environments combined with often long journeys by foot, exposure along a route is primary. As a result, this paper utilises a novel approach to model human movements in a rural setting in Africa, using a pathfinding algorithm which calculates a path between home and resource based on information about the surroundings.

Agent-based models provide a useful tool for the exploration of fine scale human movements as they, by definition, simulate the behaviour of individuals. Furthermore, the ability to incorporate spatial heterogeneity at a similarly fine scale through the incorporation of land-cover data means that the influence of an individual’s surroundings can be considered for even very short journeys. We argue that, through the implementation of an agent-based model, it is possible to estimate human movement patterns in rural settings and, thus, exposure to a given hazard, based on knowledge only of the spatial distributions of the (i) human population, (ii) resources upon which humans depend, (iii) landscape through which humans must travel in order to reach the necessary resources, and (iv) the hazard.

### Agent-based models

Agent-based models are a class of computational models for exploring a system through the simulation of the interactions between individuals using simple rules, with often quite complex, emergent behaviour [13], and have been considered a “third-way” of conducting scientific research through the incorporation of both deductive and inductive approaches [14].

The use of agent-based models (ABMs) as a unique means of capturing human systems is well documented [15]. Examples range from spatially abstract environments such as Sugarscape [16], to applications which require greater detail regarding space representation and human movement, such as carnival crowd control [17] and evacuation planning [18].

ABMs focus on interactions (agent-agent, agent-environment) in ways that other modelling techniques find difficult to capture [19]. This leads to notable success for ABMs in domains like traffic and pedestrian flow where the interaction between vehicles or people can be modelled [20].

### Calibrating ABMs

For ABMs to be predictively useful, the basic assumptions about movement rates and movement strategies must be well founded (e.g. cars move at the right speed, people fighting to get out of a burning plane move plausibly, etc.). As a result, there exists a strong motivation for developing methods for calibrating an agent’s movement strategies against data. As researchers must often accept data in the form that they are provided (e.g. census, surveys, mobile phone records, etc.) there is a need for flexible methods for comparing a process model (an initial theory of what people do, applied to a set of agents) to data from the real world in any arbitrary form. The detail captured in ABMs is particularly useful here because it makes possible the simulation of arbitrary data collection methods in the model, to match those in the real world, thus, allowing a direct comparison for calibration purposes.

In statistical model fitting, as practiced in much of empirical science, there are well established techniques (e.g., method of maximum likelihood) for deciding which of a suite of possible statistical models gives the best fit to data. However, while conceptual frameworks for spatially explicit ABMs in epidemiology have been formulated [21] and explored [22], at present, little information is available in the literature concerning how real world data can be used to calibrate simulated human movements in an epidemiological ABM. Thus, a challenge to the wider acceptance of ABMs is the lack of universally agreed methods for calibrating them against data. Given the additional issue that calibration data are not always available for ABM studies due to the issues of cost and accessibility discussed previously, ABM construction can be hindered, and this could be one of the reasons why there are relatively few studies presenting epidemiological ABMs in the literature. As a result of these factors, many ABMs are not calibrated to data at all and are left as suggestive, approximate theoretical exercises.

Recent developments in the study of spatial epidemiology have seen ABMs applied to systems involving multiple classes of agents (e.g., human, livestock, fly) to investigate disease transmission [23]. As with the examples of social systems provided above, the resolution at which movements through space and time need to be represented varies in the field of epidemiology. When considering the average flows of people at a national or continental scale over a long period of time (when investigating, e.g., the large area prevalence of a disease such as malaria) modelling the daily frequency and direction of individual’s movements may not be that useful. However, when investigating a vector-borne disease in a sparsely populated environment at a local scale, the spatial locations of hosts and vectors are likely to be of fundamental importance to disease dynamics [24]. Indeed, capturing the spatial heterogeneity of a study area in landscape epidemiology can be an integral component of a realistic disease simulation [25].

As part of an existing interest in modelling the epidemiology of Human African Trypanosomiasis (HAT, or more commonly, sleeping sickness) and Animal African Trypanosomiasis (AAT, also referred to as Nagana), we previously constructed a simplified ABM of humans, cattle and tsetse flies [26]. As sleeping sickness is a neglected tropical disease, the prospect for development of new, more effective treatments in the near future is limited, with out-of-date, difficult to administer, and partially validated treatments currently in use [27–29]. Unfortunately, where tools are available, HAT is rarely prioritised due to competing public health interests [30]. As a result, public health policy is critical, and appropriate control methods will require a greater understanding of disease dynamics [31]. ABMs used wisely can potentially lead to better informed public health policy. However, an accurately calibrated model is required to make any simulated predictions of disease prevalence sufficiently representative and, thus, potentially useful as a decision-support tool.

This paper demonstrates the potential use of ABMs as tools for generating human movement patterns, which could subsequently be used for a range of potential applications including assessment of individual exposure to biting insects such as mosquitoes and tsetse flies, contact probabilities and, thus, transmission pathways and potential. The approach depends only on an initial distribution of agents, resource locations, and a given landscape (capturing the difficulty of traversing elements in the landscape), and fairly well accepted assumptions about people’s daily resource requirements. In this study area, these include frequent trips to water and for the collection of firewood, children’s trips to school in the morning or afternoon, less frequent trips to market, and work in the fields. Thus, this paper proposes for the first time the use of ABMs to *simulate* human movements based only on widely available geospatial datasets, as an alternative to expensive *measurement* of human movements, for use in epidemiological studies. The implication is that plausible movement patterns can be generated for any similar location, with limited need for human movement data. Using extensive household survey data from the Luangwa Valley, Zambia, a method for calibrating human agent movements against real world data in epidemiological ABMs was explored, demonstrating that the ABM method proposed here is able to predict human movements accurately.

## Methods

**Fig 1:**
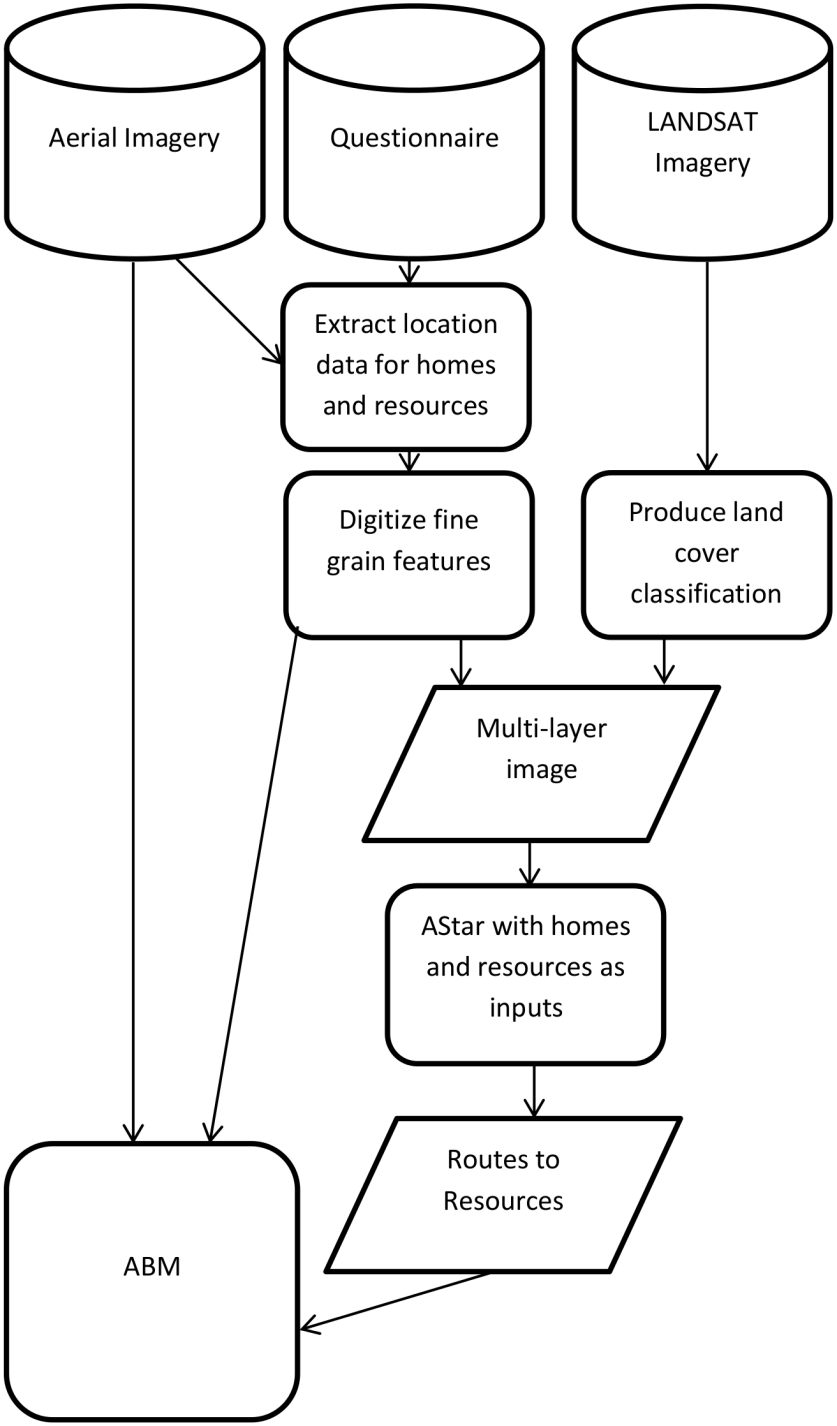
Flow chart of all methods used to produce the agent paths and movement times to calibrate to real world data.

The resources and techniques used to construct the ABM for human movement are shown in the form of a flowchart in Fig 1. The open source software Quantum GIS and Python 2.7 were used for image processing and model construction, respectively.

### Land Cover Classification and Cost Surface

When a person moves from A to B, their chosen route is often influenced by the terrain ahead of them. To incorporate this decision process into the ABM model, a cost surface of the study area was generated. Specifically, a Landsat image of the study area with a spatial resolution of 30 m was used to produce a land cover classification, dividing the region into areas of bush (high cost), farmland and cleared land (low cost). These classes allow different speeds of human movement (4 or 5 or 6 km hr^-1^) based on modifications of the movement speeds used in [32] and, therefore, need to be considered as potential facilitators or inhibitors when determining human routes to resources. Subsequently, where agent routes traverse different classes of land cover, these data can be used to define the speed at which people move through them. The incorporation of this technique may be important as exposure to different risks can be reduced or heightened depending on the precise route of human movement, but also by how long it takes for an individual to cross through one of these zones. Although it is acknowledged that variations in elevation can play a role in varying path choice and movement speed, the settled area under investigation is relatively flat with only a gentle south-to-north slope, and thus elevation was not considered in this iteration of the model as a result.

### A* Pathfinding Algorithm

Many different methods can be used to simulate human movements in an ABM. At two extremes, one could simulate linear movement by programming the agent to move along a Euclidean path between start and goal, or use an implementation of Dijkstra’s flood fill algorithm to ensure the least cost route is taken from cell to cell or pixel to pixel, regardless of how direct it is. In real world terms, the first approach simulates a person who wants to get from point A to point B as fast as possible, assuming that there are no obstacles in the way, and that the terrain underfoot is uniform, whereas the second approach simulates a person who will get from A to B eventually, at minimal cost to them (e.g. avoiding unfavourable land cover etc.).

Something more complicated than either of the above options is likely to be a better match to reality, and this requires finding a balance between the two. Indeed, while it is often important to move as directly as possible to a goal, certain areas along this direct route may be impassable or at a higher cost than the individual is willing to allow.

Although numerous pathfinding algorithms have been used in the literature, A* [33] was used in this research as it was considered to be the technique which most accurately replicates the process by which a human would devise a route to goal, while having the added advantage of being computationally economical.

By combining a Euclidean distance heuristic (i.e. the straight line route from start to goal), with the previously described cost surface, a search which resembles a ‘directed flood fill’ is produced. In this sense, while the cost surface between different pixels from start to goal remains constant, the most efficient straight line route is preferred. However, when a higher cost region is encountered along the path, the algorithm will decide whether the optimal route is to travel around the high cost area, or through it, in order to continue moving towards the goal.

### Questionnaire Data

**Fig 2:**
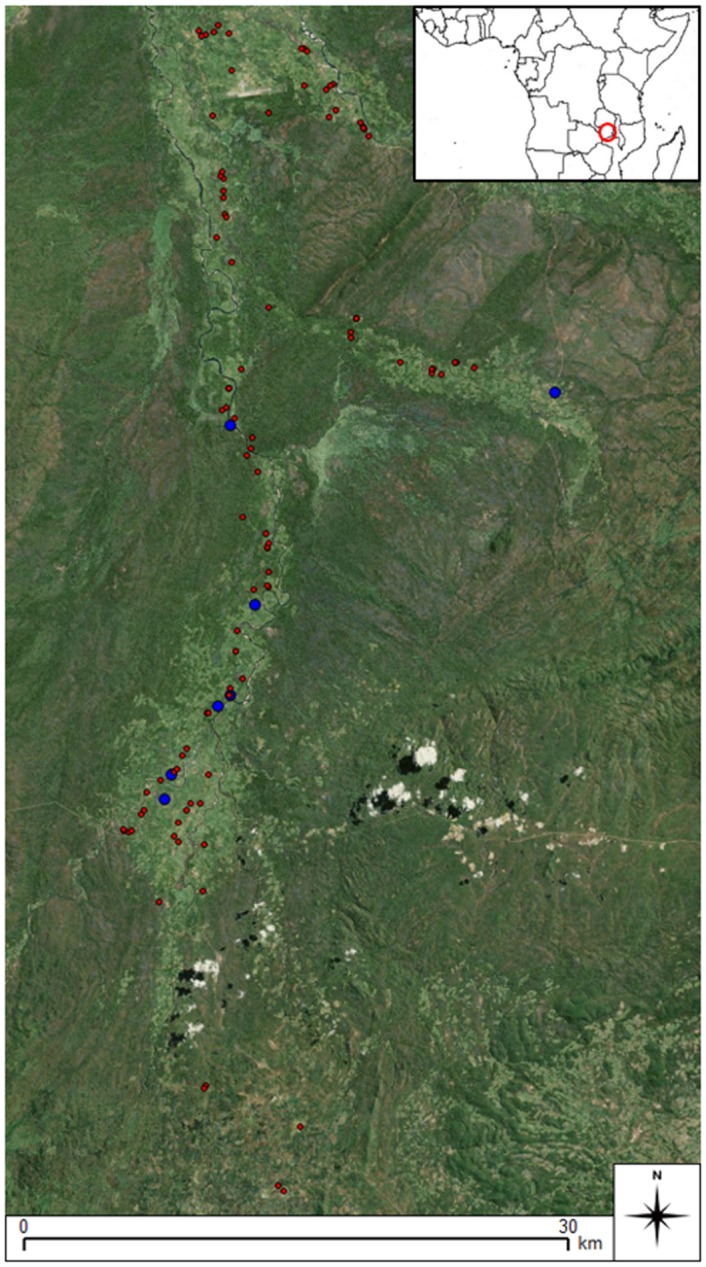
Sample village sites (red circles) and observed boreholes (blue circles) in the study area, Luangwa Valley, Zambia (Produced using Landsat 7 imagery from USGS).

Information regarding the time people take to collect domestic water was collected as part of a wider human movement questionnaire in the Luangwa Valley, Zambia, in June 2013 (S1 Appendix). The valley is an extension of the Great Rift Valley in East Africa, and lies across the Eastern and Northern Provinces of Zambia Fig 2. The survey was administered to a sample of 94 individual households. Individual-level information such as sex, age and relationship to household head was collected, along with their village role, and the amount of time an individual thought it took them to make a single journey to the water source of their choice. Information was also recorded on the frequency of these collections, including number of collections per day, and whether these occur in the morning, afternoon or evening. While the sample’s household locations were identified using GPS, along with the locations of a number of boreholes, as the data collectors could not accompany each individual on a trip to water, the exact location of different riverine water sources was not recorded.

### Multi-Layer Input Map

**Fig 3:**
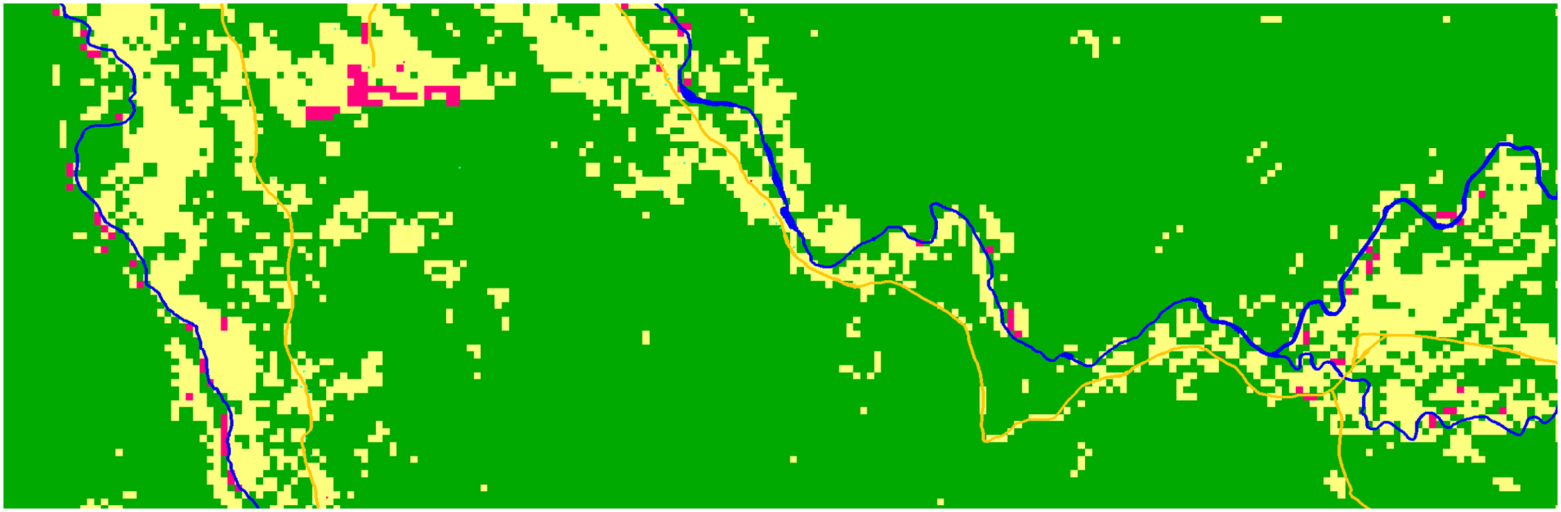
Sample section of the land classification map, with forest (green), crop (off-white), and bare land (magenta) areas highlighted. Road and river are represented by gold and blue lines respectively.

The GPS coordinates obtained for household location were overlain on fine spatial resolution Bing aerial imagery, accessed through the open layers plug-in in QGIS. The previously mentioned 30 m spatial resolution land cover classification was then overlain on this finer spatial resolution imagery, before cross-referencing and editing through the digitizing of river and road detail which was too detailed to be captured at the coarser spatial resolution. The product was a multi-layer input map for the model, comprising spatial information on household, river, road and borehole locations, along with pixels allocated to the classes of bare ground, forest and crop growing fields, at a spatial resolution of 11 m. Fig 3 shows a sample section of the land cover classification layer for the model.

### Program Description

Two separate computer programs were used to produce the observed results. Firstly, a program was written to implement multiple heuristics in the A* algorithm, producing different paths from the 94 households to different riverine and borehole water sources. This was implemented using the Iridis 3 supercomputer at the University of Southampton. Secondly, an ABM was constructed to test how long it takes agents to travel along each of these paths, given the impedance resulting from the cost surface. These programs are described in greater detail in the methodology.

## Study Area

The study area spans a sparsely populated region of the rural Eastern Province of Zambia. Villages are small (between 5 and 20 households) and inhabitants are predominantly subsistence farmers. Based on direct observations in the field, and questionnaire responses, the prominent mode of transport in the area is walking, with walkways varying from a few more established roads, to small dirt tracks or ‘desire paths’ which cannot be seen on satellite sensor imagery. The data collection area consists of a 75 km transect which starts close to Mfuwe airport in the north, and runs southwards along the Lupande River and its distributaries. Data were collected in the form of questionnaires (as described above) in Zambia from June to August in 2013 as a part of the NERC ESPA-funded Dynamic Drivers of Disease in Africa Consortium (DDDAC) research programme.

The study area is a site of interest in Trypanosomiasis research as the Luangwa valley region suffered a sleeping sickness outbreak in the 1970s. During this outbreak 241 cases were reported in a small section of the Luangwa fly belt around Isoka over a period of three years [34]. Attributed to an encroachment of the tsetse fly belt, this was a drastic increase in the number of cases, given that only 15 cases were reported in the previous three years in a larger section of the fly belt (Mpika) [35], and cases continue to be reported. In addition to such outbreaks, the ability of the low level of transmission to maintain itself for long periods is considered to be an enigma in the epidemiological field [36] and, with the Rhodesian form of the disease being a zoonosis, the significant game reservoir adds complexity to the system, with migration of hosts into the valley [37]. In the scope of this study, important environmental resources are distributed throughout the region (e.g. water) and, thus, a degree of human exposure to the hazard posed by the tsetse fly is regarded as necessary. Therefore, accurate prediction of human movement patterns may highlight areas to target with mitigation strategies in the future.

The field study gained ethical approval from ERES Converge, a Zambian private research ethics board.

## Methodology

### A* Algorithm

The land cover map introduced above was used to produce a grid of cells with values associated to their cost in terms of human movement speed. For example, road was allocated the least cost, followed by crop and bare land, with bush allocated the highest cost. While river levels are highly seasonally dependent, river cells were considered impassable in this study, reflecting river levels in the rainy season, where human exposure is likely to be higher due to more favourable conditions for the tsetse fly. Whilst beyond the scope of this study, it would be possible to adjust this assumption, varying the weight applied to river cells in order to reflect seasonal changes in how ‘passable’ the river is. Land cells that neighbour the river were collated and a random sample of 1000 of these was used as a set of potential riverine watering sites. Agents were allowed to choose randomly between the three sites closest to their village as their resource goal.

In each iteration of the model, the algorithm assessed the cost of moving to each of its eight neighbouring cells based on the summation of two factors; *g* and *h*:

$$\begin{array}{c} f_{\left(child\right)}=g_{\left(child\right)}+h_{\left(child\right)} \end{array}$$ (1)

$$\begin{array}{c} g_{\left(child\right)}=f_{\left(parent\right)}+C\alpha \end{array}$$ (2)

The *g* score represents the cost of movement between the start and the current cell, which is known and includes accurate land surface costs thus far, while the *h* score is the remaining linear distance to the goal. *C* represents the cost of moving from the current cell to the next (potential) cell, which varies depending on the land cover. *α* represents the weighting of the movement cost, with a higher value of *α* resulting in the algorithm favouring low cost cells such as road, over the most direct route.

At each iteration the optimal cell is selected from the eight neighbours, which represents the best path to take (lowest *f*(*n*)), and made into the ‘child’ of the current cell before it itself becomes the ‘current cell’ in the next iteration. This means that the algorithm creates a trail of cells that results in the path so far, which is linked by parent-child connections. By taking this approach, this also means that there could be multiple potential paths during the process. When one of these potential paths reaches the goal, the parent-child connections from goal to start are declared the best possible route, and the algorithm ends by creating a list of these cells, along with the corresponding cell land cover classes.

As the construction of these paths is a variable under investigation, how much of an impact the land cover has on the chosen path (i.e. how far will an agent travel off the Euclidean route to walk on, e.g., road instead of crop) was varied for multiple runs.

**Fig 4:**
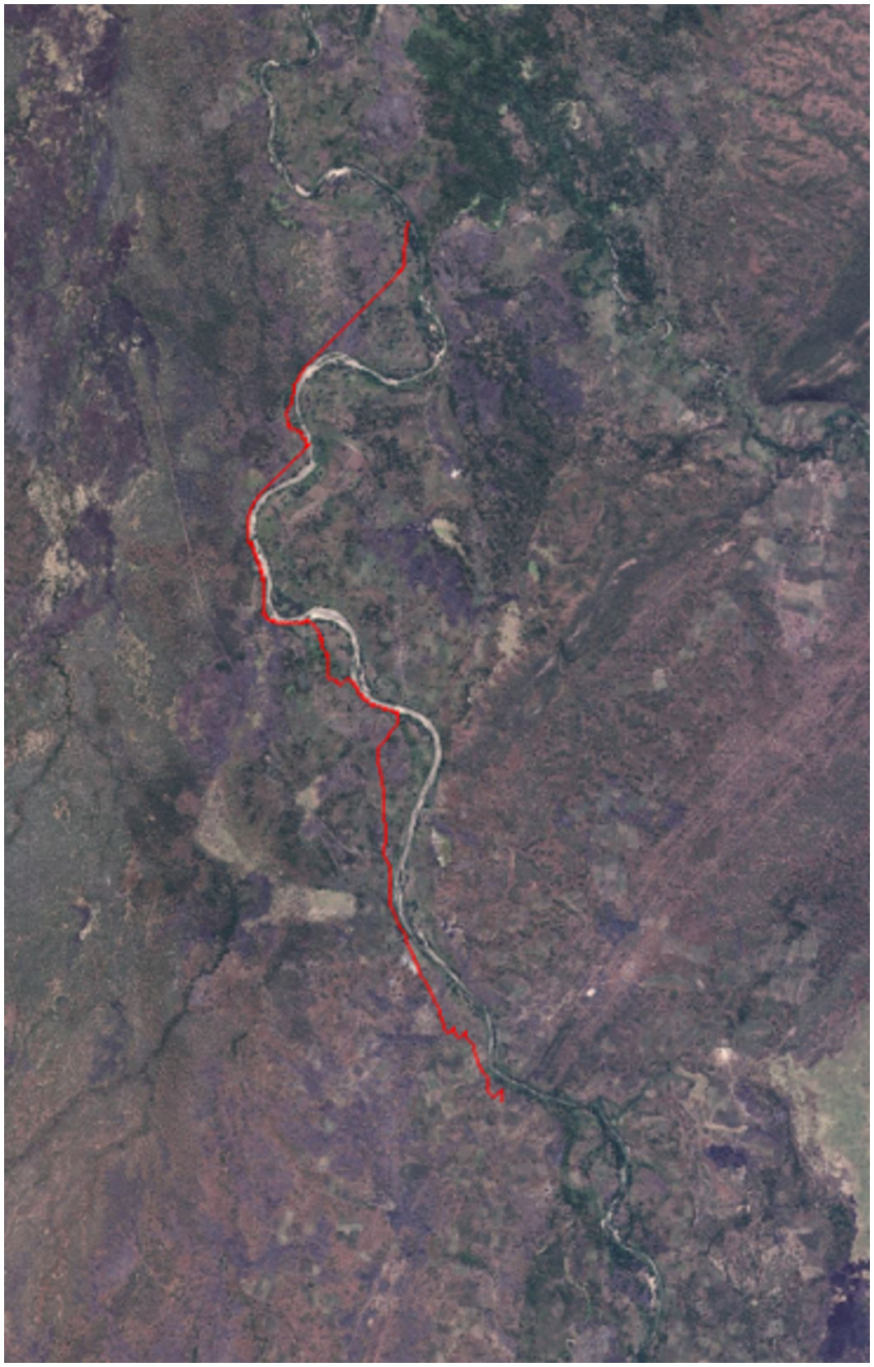
Example path produced using the A* and land classification between arbitrary points. Arbitrary points were used to emphasise how the algorithm diverts the path around a prominent obstacle; in this case, the river itself (Produced using Bing aerial imagery).

In the real world, when a person chooses a route of travel, there is often a conflict between the most direct path, and the ‘easiest’ path to the destination, often resulting in a compromise. This paper seeks to identify the appropriate balance of these two factors in this investigation by varying *α*. *α* was varied from 0–45, providing a sufficient range of weightings to identify the optimum choice. The cost (*C* in the above equation) was multiplied by *α* before adding to the *g* score in different runs to give a greater weighting to certain land covers. Therefore, as the value of *α* increases, the algorithm will increasingly favour low cost routes at the expense of direct travel. An *α* = 0 run was included as a control as this represents the Euclidean distance. *h* was multiplied by 10 in each run for computational efficiency, removing the decimal number associated with diagonal moves. Future references to the different variations of the algorithm, and the results they produce, will be by their respective *h* and *g* values in the form: *H*10*Gα*. Fig 4 shows an example path produced using the algorithm in the study area of this investigation.

### Agent-Based Model

Initially, the 11 m spatial resolution aerial imagery was made into a graphical interface to observe the subsequent simulation running using the Tkinter package in Python 2.7. The cost surface land cover information, along with the coordinates of villages, boreholes and potential watering sites were incorporated as arrays. Populations were then initialised, placing 5000 agents randomly across the villages in the simulation. A large number such as this was used so that each village would be populated and therefore a comparative simulated distribution of walk times could be produced. Iterations of the simulation were used to represent clock time (18 seconds per iteration), where an agent would move between their home village, and a watering site (either borehole, or one of the three closest riverine sites) along the previously calculated A* paths. 18 second iterations, or 4800 iteration days, were used so that the temporal resolution of the model was fine enough to include the shortest trips of agents to water, which can be less than 5 minutes. Movement speed between cells was dictated by the land cover of the cell which the agent was about to move to, so that the agent moves faster through preferable terrain such as road, and slower through the other types of land cover. The number of steps an agent takes per iteration was governed by the maximum distance an agent could travel in this period, which was defined by the land cover of the cells across this distance. The number of cells moved in one time step would be at a maximum if an agent encountered only road cells (resulting in an approximate speed of 6 km^-1^). Conversely, an agent would cover fewer cells per time step should some of these cells be, for example, bush, reducing their speed. The fewest cells covered or lowest speed would be associated with an agent encountering only forest cells. Certain combinations of cells within a particular time step may not meet the distance threshold exactly. Should the distance travelled be less than the threshold, and the difference less than a step size, a random draw was used to decide whether another step was taken in this iteration. The time taken for the agent to travel a single journey from village to the water resource goal was recorded in minutes. Return trip times were not considered in this investigation due to the form of the questionnaire data, but also due to the difficulty in quantifying the reduction in walk speed which could result from carrying a heavy load of water.

The results were divided into bins to allow a Chi-squared analysis, comparing the distributions of the simulation results and the questionnaire data. The simulation was run again for a single agent per village to allow analysis of the individual error between the simulation and real world data.

## Results

**Fig 5:**
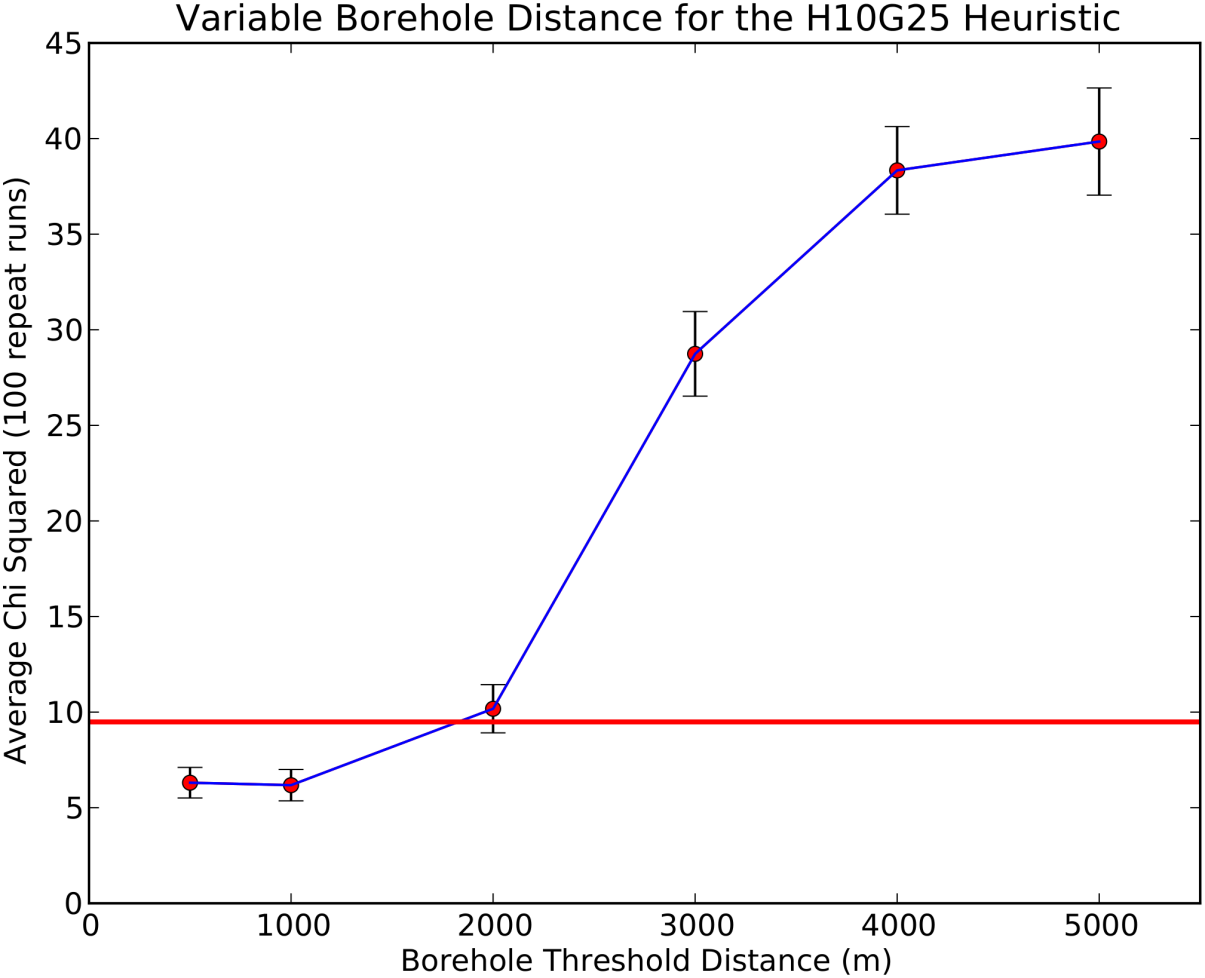
Chi-squared values and standard deviations for ten repeat runs of each borehole threshold for heuristic model H10G25. The red line shows 95% significance.

The model was run multiple times to investigate the impact of two independent variables on the trip times of agents from home to water. The first independent variable is the seven different sets of paths produced by varying the *g* score in the A* pathfinding algorithm, which are now represented in the form H10G05—H10G45. The second independent variable is a borehole threshold. The borehole threshold is defined as the distance that an agent is willing to travel to a borehole in order to collect water from this superior source, and ignore closer riverine water sources. These borehole thresholds are taken from the set 0.5, 1, 2, 3, 4, 5 km and the simulation results are compared to the observed data. A matrix of Chi-squared results for the goodness-of-fit between simulated results and the questionnaire data for these different heuristics is shown in Table 1. The results vary greatly (min = 6.54, max = 65.19), with all relationships where the Chi-square statistic is not significantly elevated (Chi-squared < 9.488, 95% conf.) being found with borehole thresholds of 2 km and below. Similarly, six of the seven scenarios where we do not observe a significant deviation between the simulation and the data were found to use a heuristic with a larger *g* weighting, where the land cover classification had a greater influence on path construction. As H10G25 has the most scenarios without significant deviation, the ABM for these paths was run for 100 repeats per borehole threshold. The results and standard deviations for these additional simulations are shown in Fig 5.

**Table 1:** Matrix of Chi-squared results for varying cost weighting and borehole distance threshold; bold indicates statistically significant (95% conf.).

**Chi-Squared**	**Borehole Threshold (km)**					
A* Input	0.5	1	2	3	4	5
H10G00 (Euc)	15.94	11.2	13.14	28.45	35.56	37.78
H10G05	9.77	11.2	**7.71**	23.29	31.29	34.11
H10G15	18.32	20.13	16.32	30.12	38.21	32.92
H10G15	15.7	14.1	14.91	35.4	43.94	47.69
H10G25	**6.54**	**7.89**	**8.73**	26.22	41.5	44.8
H10G35	10.35	**8.95**	17.31	36.4	49	65.19
H10G45	**6.64**	**6.7**	13.1	26.39	40.44	39.43

**Fig 6:**
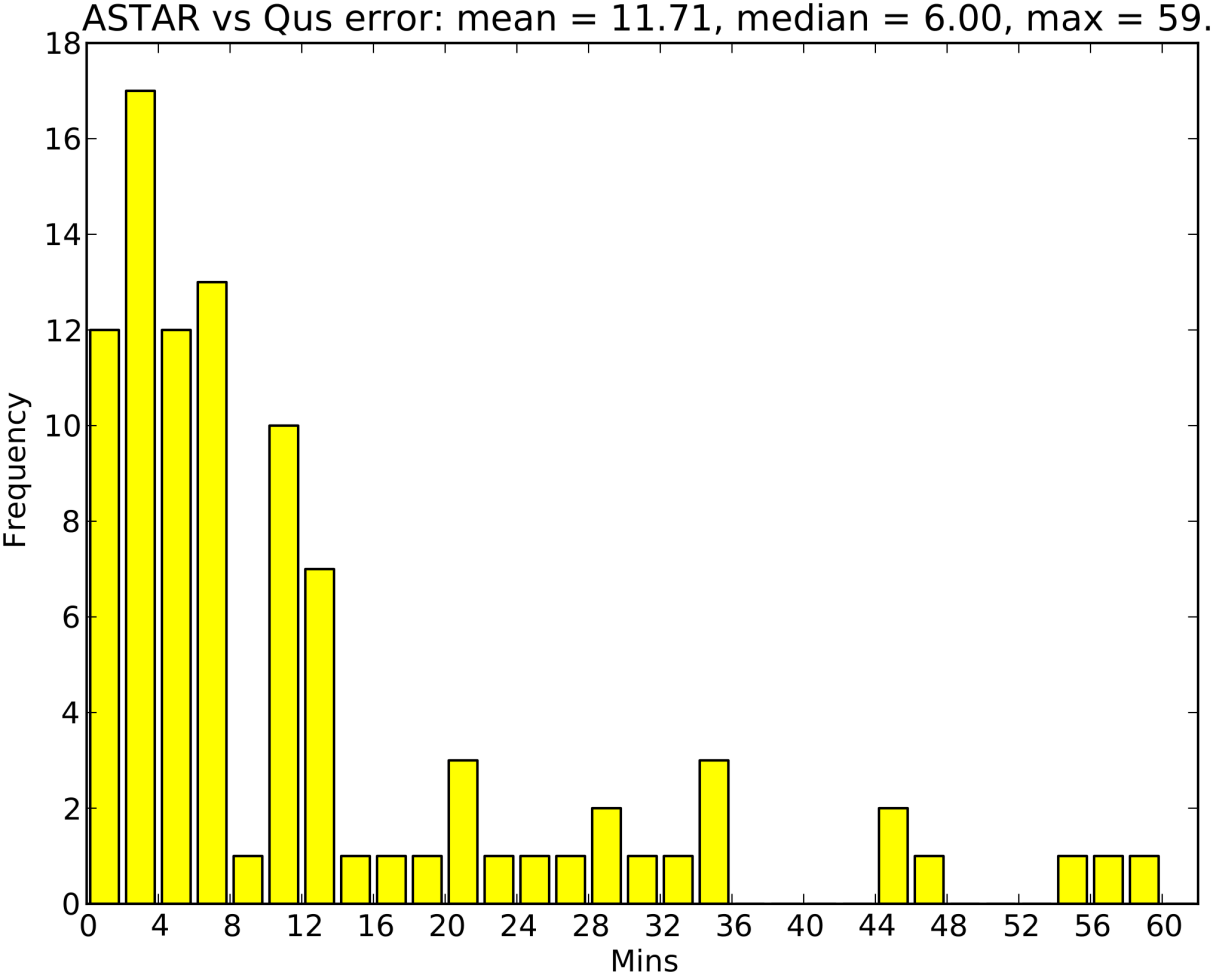
Distribution of the individual errors (difference between simulation and questionnaire results) for the H10G25 simulation, with a 1 km borehole threshold.

The model of best fit was further analysed by plotting the difference between actual walking time and the simulated walk time to water for the corresponding individual households (represented by one agent per village), allowing for more in-depth comparison through the production of an error distribution (Fig 6).

The plot has a right-skewed distribution, with 75% of the simulated walk times being within 14 minutes of the corresponding questionnaire data. The long tail of the distribution and large maximum error of 59 minutes accounts for the elevated mean individual error of 11.71 minutes.

**Fig 7:**
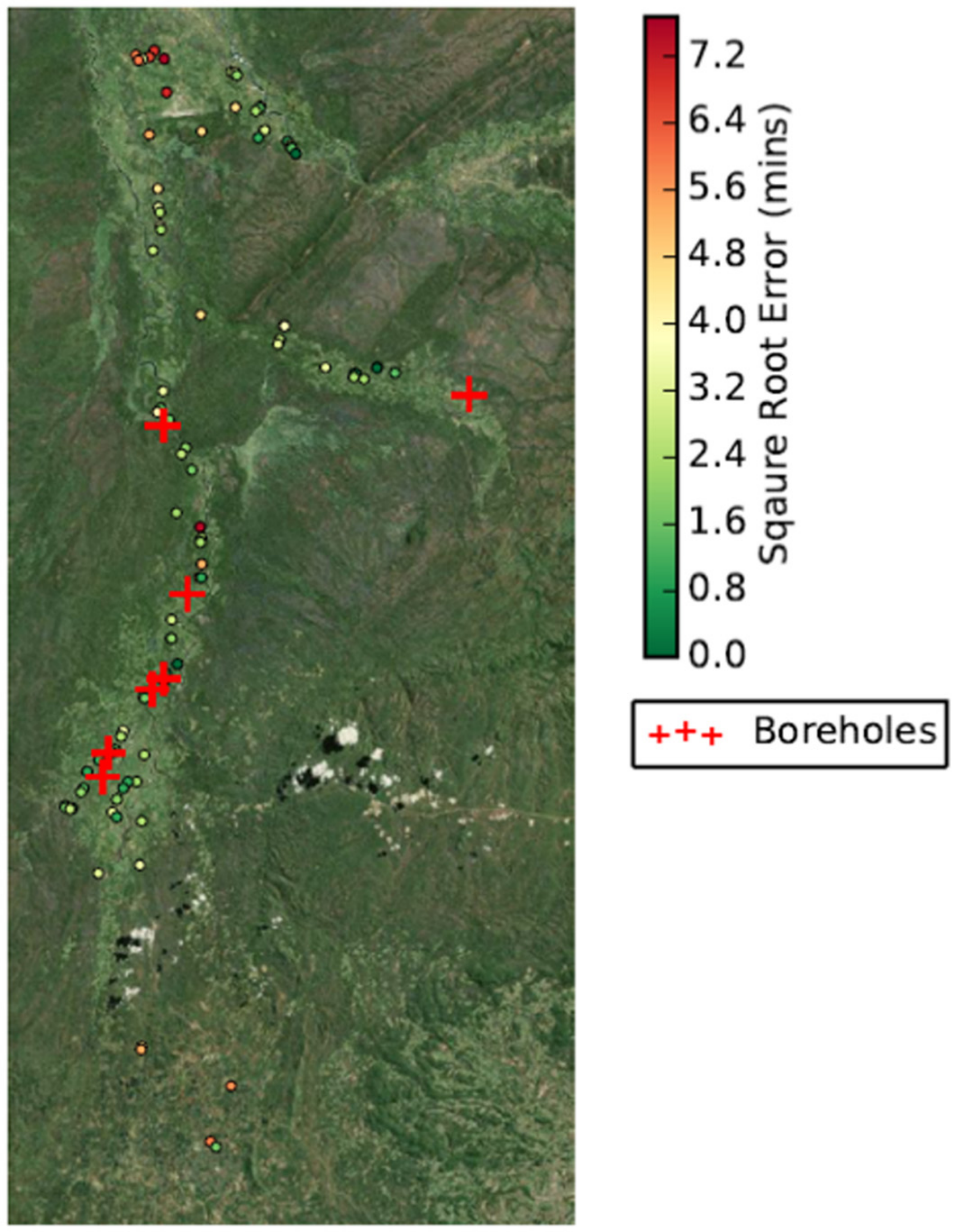
Individual errors for the single agent per village simulation using the H10G25 heuristic and 1 km borehole threshold. The square root of the errors is shown, for ease of interpretation (Produced using Landsat 7 imagery from USGS.)

To identify a possible cause for the elongated tail in (Fig 6), the spatial distribution of the error was plotted by household coordinate on an xy grid (Fig 7). Green circles indicate households with very low errors in individual walk times, red circles indicate those with larger errors, and red crosses represent the locations of known boreholes. For this figure, the square root of the error was plotted. The most obvious cluster of large errors is in the area furthest north with the households that lie close to the airport; these are located furthest from known boreholes and river sites. Households around the areas where boreholes are known to be located have smaller errors in general.

## Discussion

The optimal parameter set (the H10G25 pathfinding heuristic and a 1 km BT) appears to represent reasonable values as both parameter values lie between the fastest possible scenarios (i.e. straight line movement to the nearest water source) and the least cost to the individual with the best quality resource (i.e. movement across the most forgiving terrain to the nearest borehole water source). This reflects expectations, given our understanding of human decision-making processes and physical capabilities, and the fact that these trips to water make up only a part of an individual’s daily responsibilities. For example, the optimum pathfinding heuristic, which lies somewhere between the absolute least cost path and Euclidean movement, represents the decisions made by humans every day. Imagine a scenario where an individual walks along a country path which detours around a wooded area and continues at a similar point on the other side. Based on the time pressure of the trip, length of the detour, the quality of the path, the size of the obstacle, and the penetrability of the wooded area, an individual will likely make a decision on whether to take a short-cut through the wooded area or not. With the inclusion of the cost surface and a proportion of the A* heuristic preferring direct movement, the model simulates this sort of decision-making process. The result is a very believable set of scenarios where agents will avoid obstacles or high cost surfaces up to a point where the accumulated pixel-based cost is such that it is no longer beneficial to veer away from the direct route to goal.

Similarly, in an ideal world, all people would be able to collect their water from the preferred borehole sources. However, the Chi-squared results for low borehole thresholds suggest that this is not realistic. Again it is easy to see that, with other daily responsibilities, people are unlikely to ignore nearby riverine water sources in favour of a long (e.g., 10 km) round trip to a borehole source. The simulation instead suggests that round trips of 1 km or 2 km are the limit to how far people are willing to travel to a borehole source, potentially ignoring much closer riverine sources. This is likely to reflect not only the time pressure on the task, but also the physical limitations of carrying large vessels of water over a long distance.

Observed travel times do not significantly differ from predictions from the model using the optimal heuristic (H10G25) for 0.5 km and 1 km. The square root of individual errors suggests that the model predicts walking times accurately in the majority of villages, especially where boreholes have been identified, except for a few anomalies. However, clusters of errors form in the north and south of the transect. The uniformity of these errors in close proximity to each other suggests that a borehole or reservoir water source has been missed, particularly as these sites lie further from the river than most households in the central region. In particular, the close proximity of the red households in the north towards the airport, a notable area of infrastructure in the region, suggests that these overestimates of up to 59 minutes can be accounted for by water sources which have not been recorded. It is worth noting that the ability to visualise the errors spatially facilitates this type of diagnosis, and can be extremely helpful in supporting further model development.

An important methodological point which has arisen from this investigation is that the method used allows one to challenge or notice discrepancies between the theoretical picture suggested via the ABM, and the collected survey data. While the value of quantitative information is appreciated, an advantage of this method is that where lack of coherence between model and data arises, the data can be questioned, and possible gaps identified. Although a more accurate measurement approach would arguably be through GPS tracking of individuals throughout their daily routine, such methods come with their own potential issues, such as a lack of understanding of the technology, technical faults, misuse and financial cost. The method presented relies primarily on remotely collected data, calibrated to a short sample questionnaire which can be affordably attached to larger fieldwork studies. As a result, the method and associated model are sufficiently general to be applied to different resources, sought by different populations, in different poor, sparsely populated rural areas, given the correct satellite sensor imagery, and a small sample of real world movement times. Furthermore, possible applications of the resultant route data are varied, including the calculation of catchment areas for resources, and the production of ABMs of disease transmission.

With A* being a novel approach for such an investigation, direct comparisons would be difficult. However, through the creation of multiple versions of the algorithm, it has been highlighted that the use of A* is justified as it outperforms the null H10G00 (Euclidean equivalent). Furthermore, the additional information acquired using this approach compared with the Euclidean metric, such as fine scale deviations from the direct route and time spent on specific land classes, provide important information for epidemiological study where exposure can vary with spatial heterogeneity and, critically, where transmission depends on proximity. While this paper has presented an example of the A* algorithm which simulates human movement behaviour accurately in this case study, it should be noted that the method has been developed, and is appropriate, for sparsely populated rural areas, such as the one described, and may not be extended to urban settings without significant additional research and modification. The main concern here is the characterisation of realistic human movement patterns for use in modelling neglected tropical disease transmission systems, which impact primarily on the poorest rural populations of the developing world.

## Conclusion

This research has demonstrated that it is possible to generate movement patterns using ABMs which, in the example, are a very close match in comparison to empirical observations of travel times to the water resource. Using the ABM and commonly available geospatial datasets on population distribution, land cover and landscape resources, it was possible to impute daily activity movement patterns to the water resource for all surveyed villages in the 75 km long study transect without costly measurement as is commonly achieved, for example, through GPS, or retrospective or real-time diaries. This opens up the possibility of using the movement patterns to assess exposure to environmental hazards such as, in the present case, disease carrying biting insects. Importantly, since the ABM is process-based, it is possible to generalize the approach to other areas where geospatial data are available.

While there can be no substitute for direct and intensive measurement where the quality of information is key, the approach generated here holds promise for multiple applications, including rapid assessment where measurement is impossible, and simulation in areas that are inaccessible. Moreover, there is no gold-standard method of measuring movement patterns, and all methods are known to be at risk of large uncertainties and blunders (e.g., GPSs being loaned to siblings, being removed from the person for periods of time, etc.). Whilst the simulated walk times show a large correlation with real-world data, further cases should be explored. Nevertheless, the novel modelling approach presented here holds promise as a data fusion engine, capable of integrating measurements from multiple sources, and additional sites could be explored using the same remotely acquired data sources used in this investigation. Furthermore, as the model is process-based and not data-driven, questions concerning the data and its completeness can be identified and addressed readily.

Future research will use this study as a basis to investigate sleeping sickness transmission, using the calibrated movement model produced here to identify exposure of agents to high density fly zones, simulating contacts and infection.

## Supporting Information

S1 Appendix Questionnaire Data. Questionnaire responses and distribution for walk time to water resource.
(DOCX)
