# Supplementary material for: Exploiting Human Resource Requirements to Infer Human Movement Patterns for Use in Modelling Disease Transmission Systems: An Example from Eastern Province, Zambia
Source: PLoS One. 2015 Sep 30;10(9):e0139505. doi: 10.1371/journal.pone.0139505 (PMC4589342; doi:10.1371/journal.pone.0139505)
Supplement: S1 Appendix — Questionnaire responses and distribution for walk time to water resource. (DOCX) [file pone.0139505.s001.docx]

**2013 Zambia Data Collection - Human Movement Questionnaire Results**

The following section of text outlines the questions asked, and the subsequent responses, from the 94 households illustrated in Figure 2.

The data show that, unsurprisingly, the vast majority of the respondents collect water regularly from external water sources. The ~8% of respondents who do not collect water on a regular basis may have another member of the household who fulfils this role.

Similarly, most respondents collect water multiple times per day, and approximately half of these identify two time periods (from morning, lunchtime, afternoon and evening) when this collection takes place.

Morning and afternoon are the most popular times to collect water, and the maximum recorded single journey time to the resource was 60 minutes.

**Water Collection – (94 complete responses)**

**Personally collect water regularly?**

Yes: 114 (90.5%)

No: 10 (7.9%)

Sometimes: 2 (1.6%)

**How often do you go?**

Several per day: 99 (78.6%)

Once per day: 16 (12.7%)

Rarely: 2 (1.6%)

N/A: 9 (7.1%)

**What times?**

3 time periods given: 27 (21.4%)

2 time periods given: 66 (52.4%)

1 time period given: 24 (19%)

N/A: 9 (7.1%)

**Frequency of time period in responses:** (N.B. includes multiple responses per person)

Morning: 114

Lunchtime: 24

Afternoon: 75

Evening: 24

**Trip time to water (minutes):**

Median = 10, Mean = 12.2, Min = 1, Max = 60.
